# Knowledge, attitudes and practices of French university students towards COVID-19 prevention—are health students better?

**DOI:** 10.1371/journal.pone.0287716

**Published:** 2023-11-01

**Authors:** Elodie Alessandri-Gradt, Camille Charbonnier, Jean-Christophe Plantier, Hélène Marini, Damien Costa, Isabelle Gueit, Manuel Etienne, François Caron, Noëlle Frebourg, Guillemette Unal, Loïc Favennec, Véronique Merle

**Affiliations:** 1 Univ Rouen Normandie, Univ de Caen Normandie, INSERM UMR1311, DYNAMICURE, Rouen University Hospital, Rouen, France; 2 Department of Biostatistics, Rouen University Hospital, Rouen, France; 3 Department of Infection Control, research group "Dynamics and Events of Care Pathways" " Rouen University Hospital, Rouen, France; 4 Univ Rouen Normandie, EA7510 ESCAPE, Laboratory of parasitology-Mycology, Rouen University Hospital, Rouen, France; 5 Department of Infectious diseases, Rouen University Hospital, Rouen, France; Al-Jouf University College of Pharmacy, SAUDI ARABIA

## Abstract

During the COVID-19 outbreak in 2020, public health measures (PHM) were implemented to prevent the spread of SARS-CoV-2. At university, we wondered whether health students would be more likely to comply with these safety measures against infectious disease transmission compared to other students. Thus, we collected 1 426 university students’ responses to an online anonymous survey to describe their knowledge, attitudes and practices (KAP) of COVID-19 prevention measures and to compare the opinions and practices of health students and science students at the same university of Rouen Normandy (France). A higher proportion of science students (84.6%) compared to health students (73.9%) reported knowledge of the university’s COVID-19 protocol, p<0.001. However, the health students compared to science students reported a higher compliance with PHM at home (91.4% vs 88.0%) and at university (94.1% vs 91.1%). In a multiple regression analysis, after adjustment for age, sex and university department, factors associated with higher compliance with PHM were knowledge of the university’s COVID-19 protocol and a high perceived efficacy of PHM. A SARS-CoV-2 PCR result was not predictive of compliance with PHM. The results of this online survey in French students show a high level of knowledge and practices of COVID-19 prevention Although their performances could still be improved by training, the good results of health students regarding knowledge, attitudes and practices are encouraging as these students could be an added backup force to fight against viral pandemics.

## Introduction

In the COVID-19 pandemic context, French universities had to restrict their face-to-face teaching activities during the first national lock-down period, from 17^th^ March, 2020 to 5^th^ May, 2020. This transition to distance learning had multiple heavily negative impacts on health behaviours (eating, drinking, smoking), mental health, learning aptitudes, well-being and social networks [1–4]. The re-opening of universities, in September 2020 (the universities did not re-open at the end of the national lockdown in May 2020), was therefore necessary, but was associated with a high risk of SARS-CoV-2 transmission among students [5]. Indeed, re-opening while the SARS-CoV-2 pandemic was still ongoing represented a real challenge. In this context, the French government, supported by national health recommendations for schools [6], issued specific guidelines for universities [7], as did the USA with the department of education’s COVID-19 handbook [8]. The safe re-opening of universities relied on compliance with public health measures (PHM): mask wearing, frequent hand washing, physical distancing, adequate ventilation of premises, hygiene behaviours (coughing and sneezing in one’s elbow, using a single-use handkerchief, alcoholic hand gel, etc.), SARS-CoV-2 testing, contact tracing and isolation/quarantine for COVID-19 cases.

These control measures of SARS-CoV-2 transmission were implemented by French universities in September 2020. The French government set up and regularly updated a COVID-19 prevention webpage, according to the latest indications from national protocols [9]. Nevertheless, in the early weeks after re-opening, national epidemiological reports notified a greater increase in SARS-CoV-2 incidence in the 15–44 year olds age group than in other age groups. The highest incidence rate (200 per100 000 inhabitants) was observed in the 15–19 year olds age group and was three-fold higher than in children of less than 15 years old [10]. Indeed, at week 39 of 2020, 20% of the 2,830 SARS-CoV-2 clusters in France, since the onset of the outbreak, concerned school and university students, the second most represented community, just after business companies (25%). These results suggested a suboptimal compliance with PHM to stop the transmission of the virus in the context of a potentially higher physical contact due to social networking in this population, including mixing with lots of people of their age [11].

Among university students, health students should be more sensitive to PHM, because they receive specific teaching about safety measures against infectious disease transmission. If working at the hospital, they are also involved in the prevention of nosocomial infections and good practice of hygiene. Therefore, we postulated that health students would have better knowledge and better practices of PHM compared to students from other fields of study, such as science. Indeed, previous studies in several countries highly impacted by COVID-19 have already demonstrated the major role of healthcare workers in the control of SARS-CoV-2 spread [12–14].

Thus, the aim of our study was to describe the knowledge, attitudes and practices (KAP) of university students towards PHM, to understand their potential difficulties to carry them out, and to compare the attitudes and practices of health students and science students at the same university of Rouen Normandy (France).

## Method and population

### Population

In 2020, approximately 32,000 students were registered at Rouen Normandy University from bachelor graduate to doctorates. Around 300 academic courses are available from different fields of study, but the most popular are science and Health. In 2020, the Science campus had 4,626 students registered in biology, mathematics, chemistry, physics or computer sciences. The science campus is located on the outskirts of the city of Rouen. In 2020, the health campus had 3,210 students registered in medicine, pharmacy, midwifery or speech therapy. The health campus is located in the city centre, next to the university hospital. The two campuses are 6 kilometres away from each other and the students do not usually share the same university facilities, cafeteria, library, fitness centre, etc. Although each campus has its own dean, both campuses are linked to the same university: Rouen Normandy University. Concerning healthcare and prevention, Rouen Normandy University has a specific department divided into two structures: one for the students, the other for the staff.

All health and science students of Rouen Normandy University were eligible for the survey.

### Study design

First, a descriptive analysis of the knowledge and attitudes on PHM was performed among two populations of students (health students and science students) and adjusted for sex, age and university department. Second, we specifically analysed students’ practices of two PHM (hand washing and facial mask use) generally recognised as simple and efficient barriers against SARS-CoV-2 transmission. We compared the populations of students (health versus science), their environments (university and home, hospital if applicable for health students). Finally, we searched for a possible association between the perceived efficacy of PHM and the level of compliance with PHM in practice.

### Online survey

An anonymous survey was created and published online on the Rouen Normandy University website (S1 Appendix). Respondents were asked not to communicate personal data in their comments that could lead to their identification. We received authorisation from Rouen Normandy University’s data protection officer.

The LimeSurvey tool was used for the creation and distribution of the survey through university social networks and a weekly newsletter. In addition, to obtain a high rate of responses, an individual e-mail was sent to the students of the studied populations. The survey was available online for 2 months, from 22^nd^ January, 2021, to 19^th^ March, 2021.

The questionnaire included 18 questions (4 additional questions if students worked in hospital) concerning knowledge, attitudes and practices of PHM:

i) students’ knowledge of the content of the university’s COVID-19 protocol;
ii) students’ attitudes regarding the efficacy of 13 PHM to reduce SARS-CoV-2 transmission, assessed via a -point Likert scale (ineffective/poorly effective/moderately effective/highly effective/do not know), their attitudes regarding 8 fixed responses about the application of potential restrictions of PHM;
iii) students’ self-reported practices regarding frequency of use and compliance with PHM, both assessed via a 4-point Likert scale (respectively, systematically/regularly/rarely/never and perfect /rather good/ rather bad/not at all).

In addition, students could provide unlimited-choice responses regarding the application of potential restrictions of PHM or an eventual molecular diagnostic test by Polymerase chain reaction (PCR) assay they would have had. The academic level of students (bachelor, masters or year of study) was not collected.

### Statistical analysis

All statistical analyses were realized with R 4.1.2 software. Main characteristics were computed for all 1426 responders. When adjusted on sex, multiple regression analyses were computed on the subset of 1412 individuals with specified male or female gender information. Ordinal values were approximated by quantitative values on a 1 to 4 linear scale. Hand wash and mask perceived efficacies were summed up into a single quantitative score of “perceived efficacy” on a 1 to 16 scale. When necessary, declared practices were dichotomized into “high level of compliance (perfect or rather good) vs. low level of compliance (rather bad and not at all. Health and science students’ categorical characteristics were compared using chi-square tests. Perceived efficacies among health and science students were compared using a Student t-test with unequal variances (Welsch’s test).

Graphical representations of perceived efficacies of PHMs were drawn using the *radarchart* function of the *fmsb* package. Age, Department and Gender effects on these perceived efficacies were tested within a multiple linear regression framework for each of the PHMs. For this group of analyses, type-I error rate was controlled by accounting for 39 multiple tests (13 PHMs times 3 covariates) and using a p-value threshold of 0.0013 for significance.

Determinants of knowledge, attitudes and practices were analyzed with linear and logistic multiple regressions, with consistent adjustment for age, sex and university department, and progressive inclusion of knowledge and attitudes among covariables. Type-I error rate was controlled by accounting for each of the 23 multiple tests presented in Table 3 and using a p-value threshold of 0.0022 for significance.

Reasons for low compliance were summarized among students that declared a low compliance with PHMs, at university and at home separately. Proportions of students invoking each specific reason at university and at home were compared using a chi-squared test. Type-I error rate have been controlled by accounting for each of the 8 tests, with a p-value threshold of 0.00625 for significance.

## Results

Less than a quarter of registered students from health and science courses (respectively 23.4% and 14.5%) completed the survey. Their characteristics are described in Table 1. Respondents were mainly female but with a higher proportion of health students (74.5%) than science students (58.9%). Near a half of the survey respondents had previously performed a SARS-CoV-2 PCR test, with a significant difference between students (56.3% [52.6%-59.8%]) compared to sciences students (38.0% [34.3%-41.8%], chi-square test, p<0.001). The most frequent indication reported for this test was “having had a contact with a confirmed case of SARS-CoV-2 infection”.

**Table 1:** Main characteristics of the studied population.

		Health students (n = 3 210)	Science students (n = 4 626)	TOTAL (n = 7 830)
Students who completed the survey (%)		752 *(23*.*4)*	674 *(14*.*5)*	1426
Students involved in hospital care *(%)*		284 *(37*.*7)*		
	<20 years	346 *(46*.*0)*	216 *(32*.*0)*	
Median age range *(%)*	20–25 years	377 *(50*.*1)*	433 *(64*.*2)*	
	>25 years	29 *(3*.*9)*	25 *(3*.*8)*	
Sex (N, available)		752	660	1412
Female (%)		560 (74.5)	389 (58.9)	949 (67.2)
Male (%)		192 (25.5)	271 (41.1)	463 (32.8)
SARS-CoV-2 PCR done*(%)*		423 *(56*.*3)*	256 *(38*.*0)*	679 *(47*.*3)*
Positive results *(%)*		68 *(16*.*0)*	31 *(12*.*1)*	99 *(14*.*5)*

Knowledge of the university’s COVID-19 protocol, and declared compliance with PHMs are described in Table 2. Overall, more than 70% of the responders declared to know the existence of the protocol and its contents. Sciences students reported a higher knowledge of the existence of the university COVID-19 protocol (84.6% [81.6%-87.2%]) than health students (73.9% [70.6%-77.0%], p<0.001) and more frequently declared to have read its content (76.6% [73.2%-79.6%] vs 66.4% [62.9%-69.6%], p<0.001).

**Table 2:** Knowledge of the university’s COVID-19 protocol and declared compliance with public health measures (PHMs) among respondents.

		Health students (n = 752)	Science students (n = 674)	p-value	TOTAL
Knowledge of the university’s COVID-19 protocol *(%)*		556 *(73*.*9)*	570 *(84*.*6)*	<0.00001	1123 *(78*.*7)*
Ever read it *(%)*		499 *(66*.*4)*	516 *(76*.*6)*	0.00002	1015 *(71*.*1)*
Perceived efficacy score (mean ± sd)		14.5 ± 1.6	13.9 ± 2.2	<0.00001	
High level of compliance with PHMs %	At home	91.4	88.0	0.04434	
	At university	94.1	91.1	0.03485	
	At hospital	95.8			

**Fig 1:**
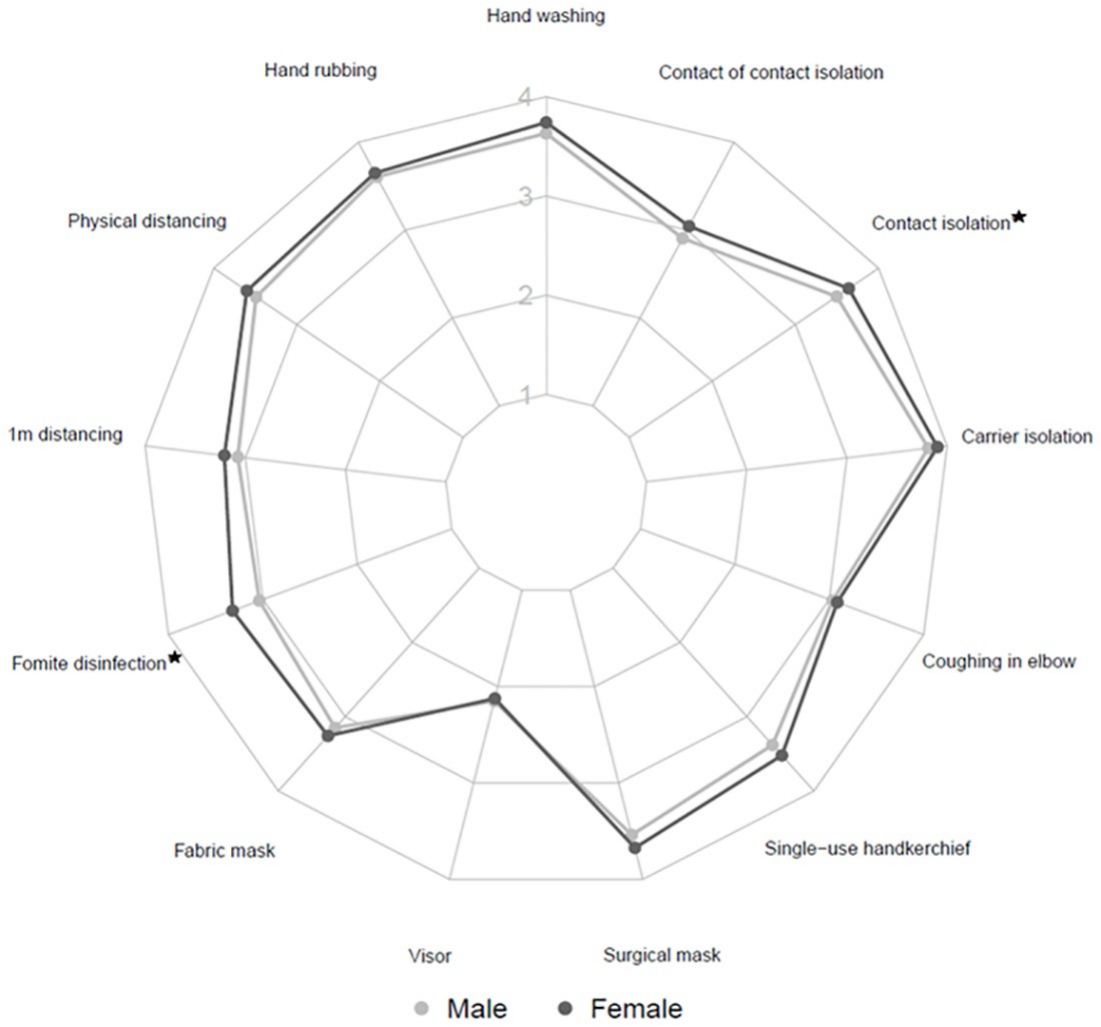
Radar showing the level of perceived efficacy of PHM items, according to sex (Male/Female). ★ effect above the threshold of test significance.

**Fig 2:**
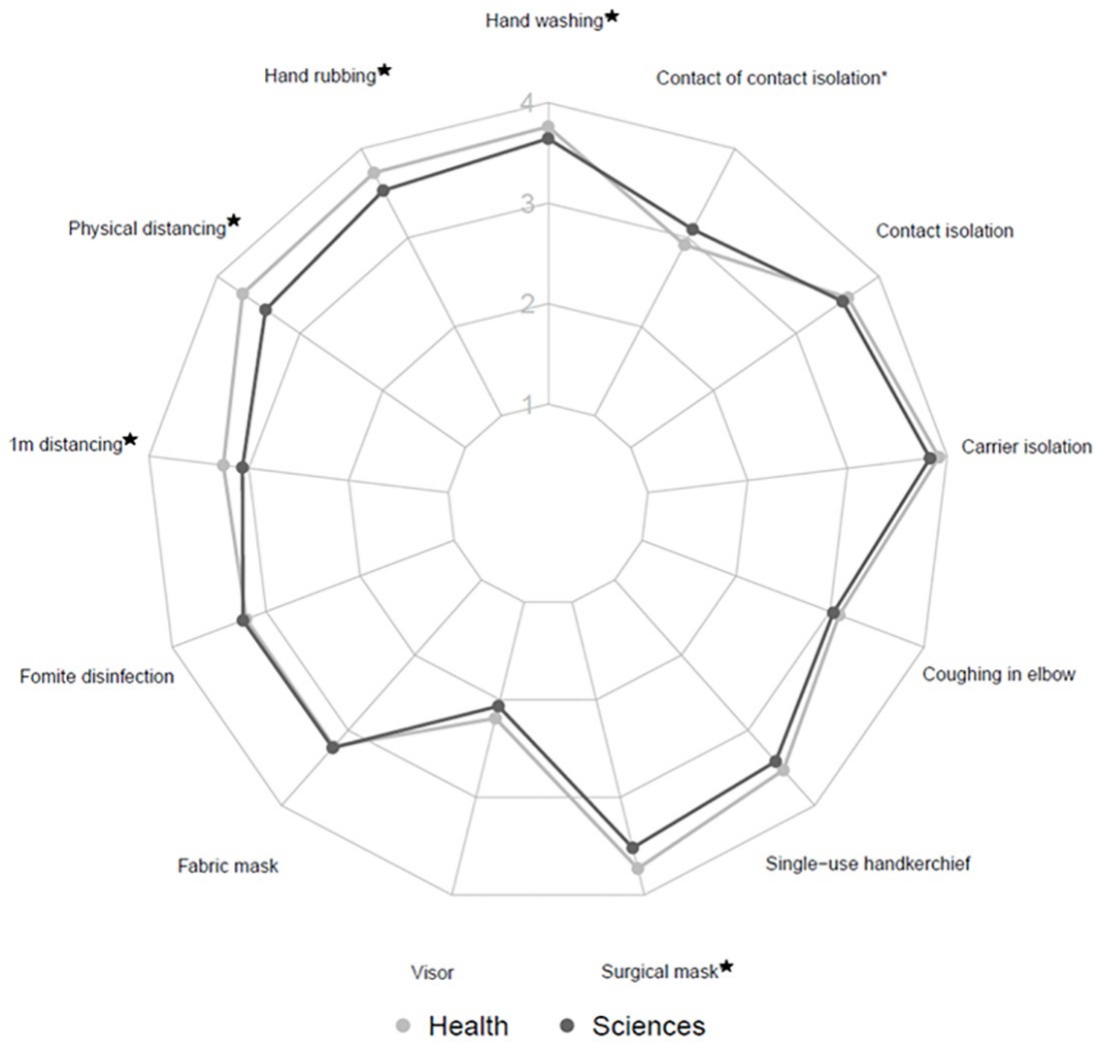
Radar showing the level of perceived efficacy of PHM items, according to university department (Health/Science). ★ effect above the threshold of test significance.

Concerning the perceived efficacy of different PHM items, the calculated score was not different between health and sciences students. The details of the perceived efficacy of each PHM item are presented in Figs 1 and 2. The students tended to attribute a lower efficacy to two PHM items (visor and “contact of contact” isolation) whatever their sex (Fig 1) or field of study (Fig 2). Health students, whether involved in hospital care or not, systematically assigned a higher level of efficacy to all PHM items than science students, except for one item: “contact of contact isolation” (p<0.001).

Although all the students, irrespective of their department of studies, declared a high level of compliance with PHM, health students declared significantly higher compliance than science students, both at home (91.4% [89.1%-93.2%] vs 88.0% [85.2%-90.3%], chi-square test, p = 0.04434) and at university (94.1% [92.2%-95.7%] vs 91.1% [88.6%-93.1%], chi-square test, p = 0.03485 (Table 2).

Factors associated with knowledge, attitudes and practices, after adjustment for potential confounders, are listed in Table 3. Fitting a logistic model simultaneously on age, sex and university department, factors associated with knowledge of the university’s COVID-19 protocol were: i) age, with less-than-20 years old students declaring a better knowledge of the protocol existence than older students (OR = 0.87 [0.83; 0.91] for [20–25[years old students, p<0.001); ii) university department, with a better knowledge for sciences students (1.14 [1.09; 1.20], p<0.001).

**Table 3:** Knowledge, attitudes and practices among respondents.

	Knowledge		Attitudes		Practices			
	Protocol existence and content		Perceived efficacy (1–16)		High level of compliance at university		High level of compliance at home	
	OR	p-value	β coefficient	p-value	OR	p-value	OR	p-value
**Age (years)**								
*Less than 20*	*ref*				*ref*		*Ref*	
20–25	**0.87 [0.83; 0.91]**	**<0.0001**	-0.07 [-0.30; 0.15]	0.5256	0.99 [0.96; 1.02]	0.3851	0.98 [0.95; 1.02]	0.3836
25 or older	*0*.*83 [0*.*74; 0*.*94]*	*0*.*0041*	-0.51 [-1.05; 0.03]	0.0656	1.06 [0.99; 1.14]	0.1080	1.04 [0.95; 1.13]	0.3936
**Sex**								
*Male*					*Ref*		*Ref*	
Female	1.04 [0.99; 1.09]	0.1481	*0*.*24 [0*.*01; 0*.*47]*	*0*.*0449*	0.99 [0.96–1.02]	0.4327	1.03 [0.99; 1.07]	0.0723
**University department**								
*Health*					*Ref*		*Ref*	
Sciences	**1.14 [1.09; 1.20]**	**<0.0001**	**-0.44 [-0.66; -0.22]**	**<0.0001**	0.98 [0.95–1.01]	0.2247	0.99 [0.96; 1.03]	0.7278
**Knowledge of protocol**					**1.06 [1.03; 1.10]**	**0.0004**		
**Perceived efficacy**					**1.02 [1.01; 1.03]**	**<0.0001**	**1.04 [1.02; 1.04]**	**<0.0001**
**Past Covid-19 status**								
*No PCR test*					*Ref*		*ref*	
PCR negative test					1.00 [0.97; 1.03]	0.9989	0.98 [0.95; 1.02]	0.3689
PCR positive test					1.00 [0.95; 1.07]	0.8756	0.96 [0.89; 1.02]	0.1940

A linear regression of the score combining hand washing and facial mask use perceived efficacies on age, sex and university department showed that students from science attributed a lower efficacy (-0.44 [-0.66; -0.22] on average, p<0.001) to these two PHM items than health students. Women reported a higher efficacy than men (+0.24 [0.01;0.47] on average, p = 0.0449), although the difference did not reach significance after correction for multiple testing. Of note, in a supplementary analysis we found that health students involved in hospital care attributed a higher efficacy (+0.38 [0.04; 0.73], p = 0.02391 after adjustment for age and sex) to PHMs than other health students.

The factors associated with high compliance with PHMs at university were: knowledge of the university’s COVID-19 protocol (OR = 1.06 [1.03; 1.10], p = 0.0004) and a high perceived efficacy of hand washing and facial mask use (OR = 1.02 [1.01; 1.03], p<0.001). At home, the only factor associated with high compliance with PHMs was their perceived efficacy (OR = 1.04 [1.02; 1.04], p < 0.001). Women tended to report higher compliance with PHMs at home, without reaching nominal significance (OR = 1.03 [0.99; 1.07], p = 0.0723). Of note, a SARS-CoV-2 PCR result was not a predictive factor of PHM compliance at university, or at home.

**Fig 3:**
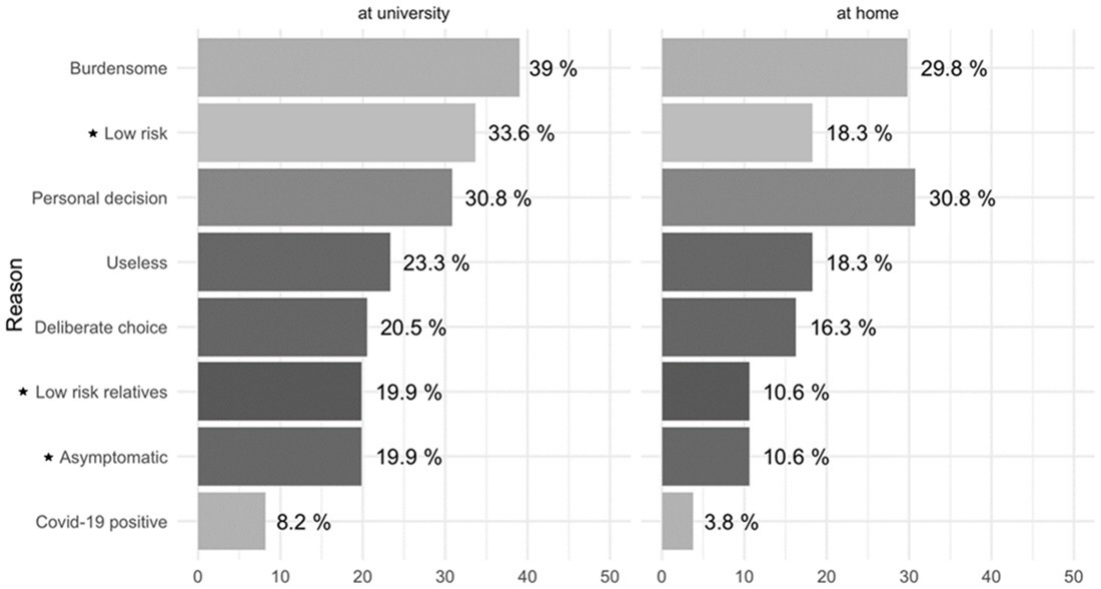
Frequency of the reasons given for non-compliance with PHMs, among respondents, at home and at university. ★ effect above the threshold of test significance.

The reasons (among 8 propositions) given by the students who reported low compliance with PHMs at university or at home (N = 128) are presented in Fig 3. An average of 2.0 reasons were given by each student that declared low compliance with PHMs at university compared to 1.4 reasons given on average for low compliance at home. At university, the main reason, namely that “PHMs are burdensome”, was given by 39% of students. The following two reasons, chosen by almost a third of non-complying students, were: “I am at low risk regarding SARS-CoV-2 infection” and “This is a personal decision”. The following four reasons were given by one out of five students: “PHMs are useless”, “This is a deliberate choice”, “I am surrounded by low-risk relatives”, “I am asymptomatic”. Finally, “I have already been Covid-19 positive” was the least given answer, by only 8.2% of students. At home, two main reasons were given by about 30% of students, namely that “PHMs are burdensome” and “This is a personal decision”. The following three reasons were “I am at low risk regarding SARS-CoV-2 infection”, “PHMs are useless” and “This is a deliberate choice”. Three reasons were less given for low compliance at home, compared to reasons for low compliance at university, at nominal significance: “I am at low risk” (p = 0.0074), “I have low-risk relatives” (p = 0.04841) and “I am asymptomatic” (p = 0.05484).

## Discussion

To our knowledge, this knowledge/attitude/practice study was the first one conducted among French students towards COVID-19 prevention.

Some studies on this topic were mainly conducted in populations with a high heterogeneity in terms of age, education and life income [13, 15–18]. Even if conducted in a more restricted profile of people, like healthcare workers, similar disparities between responders were still present [12–14]. All of these studies demonstrated that a higher educational degree was systematically associated with better COVID-19 prevention and control practice. In our study, a more homogeneous population, despite a heterogeneity of level, enabled us to search for other predictive factors of good knowledge and compliance. Indeed, we compared a population of university students, equally distributed between health students and science students, with the same bachelor degree and with a statistically meaningful number of respondents (> 1,400). Our results showed an overall high proportion (>70%) of students with knowledge of the university’s COVID-19 protocol and also a high percentage of declared compliance with PHM (>88%) regardless of the field of study However, medical students declared a higher compliance to PHM than non-medical students from the Sciences campus, both at home and at university, even though the premises of the two faculties allowed the same distancing and were supplied in the same way with distributors of alcoholic hand gel. The independent predictive factor of high compliance with PHM was a high confidence in the perceived efficacy of PHM. Two surveys previously reported higher compliance in medical students [14, 18].

Our results showed that health students were more convinced of hand washing, facial mask use and social distancing than science students. The fact that health students were more sensitive to PHM, because of training related to global hygiene measures of prevention in hospital, even before the COVID-19 pandemic, may explain this result, as it has been reported in previous studies that a greater mask use increases intentions to wear a mask [19, 20].

Despite the deterioration of students’ life conditions during COVID-19 (increase of precarity, depression, alcohol and drug consumption, eating disorders, etc.) [21], our survey suggests that they did not drop out with prevention. We have demonstrated the capacity of adaptation of students to the university’s COVID-19 protocol, whatever the university department. Students also reported higher compliance with mask wearing and hand washing at university than at home. Only 9% of respondents (N = 128) reported low compliance with PHM. When low compliance was reported at home, the reasons given were quite subjective like “it is burdensome”, “it is a personal decision”). Conversely, when low compliance was reported at university, students reported more rational reasons (“I am at low risk”, “I have low risk relatives”, “I do not have symptom”), as if the social impact of their behaviour is still of concern. Furthermore, while pandemic had a severe impact on the social concerns of the students (such as lack of socialization, stress, anxiety, and lack of motivation in attending virtual classes), university support significantly affects students’ social concerns [22].

There were some limitations to our study. All respondents were volunteers, who gave their own opinion. This may have led to an overestimation of “good practices”. However, the fact that anonymity was guaranteed probably helped students to answer sincerely. This was a cross-sectional study, so we would not have been able to show a causal link between the result of a PCR test and a specific behaviour, even if health students had an easier access to PCR tests. Furthermore, the survey was created by the health department of Rouen Normandy University and managed by the web communication of the university. As a result, despite anonymity, positive behaviours may have been overexpressed by respondents, leading to a bias of response. Our survey was restricted to two campuses, health and science, chosen because they have science courses in common. However, it would have been interesting to conduct a survey in students from literature, arts or law courses.

## Conclusion

The results of this online survey conducted in French students showed a high level of knowledge and practices regarding COVID-19 prevention. Although their performance could still be improved by training, the good results of health students as regards knowledge, attitudes and practices is encouraging as these students could be an added backup force to fight against viral pandemics.

In addition, this survey confirms that in university students, the higher perceived relevance of recommendations is associated with better declared compliance. It should lead professionals in charge of disseminating these recommendations among students, to explain in details during training sessions, the rationale for each recommendation. For health students in particular, we suggest that training could draw parallels with known infectious diseases (according to student’s curriculum) and their associated preventive measures.

## Supporting information

S1 Appendix Survey questionnaire. (PDF)

## References

[1] Flaudias V, Iceta S, Zerhouni O, Rodgers RF, Billieux J, Llorca PM, et al. COVID-19 pandemic lockdown and problematic eating behaviors in a student population. J Behav Addict. 2020 Oct 12;9(3):826–35. doi: 10.1556/2006.2020.00053 32976112 PMC8943668

[2] Tavolacci MP, Wouters E, Van de Velde S, Buffel V, Déchelotte P, Van Hal G, et al. The Impact of COVID-19 Lockdown on Health Behaviors among Students of a French University. Int J Environ Res Public Health. 2021 Apr 20;18(8):4346. doi: 10.3390/ijerph18084346 33923943 PMC8072635

[3] Charbonnier E, Le Vigouroux S, Goncalves A. Psychological Vulnerability of French University Students during the COVID-19 Pandemic: A Four-Wave Longitudinal Survey. Int J Environ Res Public Health. 2021 Sep 15;18(18):9699. doi: 10.3390/ijerph18189699 34574623 PMC8465825

[4] Bourion-Bédès S, Tarquinio C, Batt M, Tarquinio P, Lebreuilly R, Sorsana C, et al. Psychological impact of the COVID-19 outbreak on students in a French region severely affected by the disease: results of the PIMS-CoV 19 study. Psychiatry Res. 2021 Jan;295:113559. doi: 10.1016/j.psychres.2020.113559 33189368 PMC7644189

[5] Benneyan J, Gehrke C, Ilies I, Nehls N. Community and Campus COVID-19 Risk Uncertainty Under University Reopening Scenarios: Model-Based Analysis. JMIR Public Health Surveill. 2021 Apr 7;7(4):e24292. doi: 10.2196/24292 33667173 PMC8030657

[6] Covid-19: doctrines à appliquer dans les milieux scolaire et universitaire et pour l’accueil collectif des mineurs pour la rentrée de septembre 2020 [Internet]. [cited 2022 Dec 9]. https://www.hcsp.fr/Explore.cgi/AvisRapportsDomaine?clefr=877

[7] circulaire_orientations_rentree_MESRI_20200907.pdf [Internet]. [cited 2022 Dec 9]. https://services.dgesip.fr/fichiers/circulaire_orientations_rentree_MESRI_20200907.pdf

[8] reopening-3.pdf [Internet]. [cited 2022 Dec 9]. https://www2.ed.gov/documents/coronavirus/reopening-3.pdf

[9] Le protocole sanitaire de l’année universitaire 2021–2022 [Internet]. Gouvernement.fr. [cited 2022 Dec 9]. https://www.gouvernement.fr/actualite/le-protocole-sanitaire-de-l-annee-universitaire-2021-2022

[10] SPF. COVID-19: point épidémiologique du 1er octobre 2020 [Internet]. [cited 2022 Dec 9]. https://www.santepubliquefrance.fr/maladies-et-traumatismes/maladies-et-infections-respiratoires/infection-a-coronavirus/documents/bulletin-national/covid-19-point-epidemiologique-du-1er-octobre-2020

[11] Cipriano LE, Haddara WMR, Zaric GS, Enns EA. Impact of university re-opening on total community COVID-19 burden. PLOS ONE. 2021 août;16(8):e0255782. doi: 10.1371/journal.pone.0255782 34383796 PMC8360395

[12] Jemal B, Aweke Z, Mola S, Hailu S, Abiy S, Dendir G, et al. Knowledge, attitude, and practice of healthcare workers toward COVID-19 and its prevention in Ethiopia: A multicenter study. SAGE Open Med. 2021 Jul 29;9: 20503121211034389. doi: 10.1177/20503121211034389 34377469 PMC8327227

[13] Kumar R, Singh V, Mohanty A, Bahurupi Y, Gupta PK. Corona health-care warriors in India: knowledge, attitude, and practices during COVID-19 outbreak. J Educ Health Promot. 2021 Feb 27;10:44. doi: 10.4103/jehp.jehp_524_20 34084791 PMC8057180

[14] Zhong BL, Luo W, Li HM, Zhang QQ, Liu XG, Li WT, et al. Knowledge, attitudes, and practices towards COVID-19 among Chinese residents during the rapid rise period of the COVID-19 outbreak: a quick online cross-sectional survey. Int J Biol Sci. 2020 Mar 15;16(10):1745–52. doi: 10.7150/ijbs.45221 32226294 PMC7098034

[15] Ferdous MZ, Islam MS, Sikder MT, Mosaddek ASM, Zegarra-Valdivia JA, Gozal D. Knowledge, attitude, and practice regarding COVID-19 outbreak in Bangladesh: An online-based cross-sectional study. PLOS ONE. 2020 Oct 9;15(10):e0239254. doi: 10.1371/journal.pone.0239254 33035219 PMC7546509

[16] Yoseph A, Tamiso A, Ejeso A. Knowledge, attitudes, and practices related to COVID-19 pandemic among adult population in Sidama Regional State, Southern Ethiopia: A community based cross-sectional study. PLoS ONE. 2021 Jan 29;16(1):e0246283. doi: 10.1371/journal.pone.0246283 33513211 PMC7846007

[17] Shah SU, Xiu Ling Loo E, En Chua C, Sen Kew G, Demutska A, Quek S, et al. Association between well-being and compliance with COVID-19 preventive measures by healthcare professionals: A cross-sectional study. PloS One. 2021;16(6):e0252835. doi: 10.1371/journal.pone.0252835 34097719 PMC8183980

[18] Wassif GO, Din DAGE. Relationship between knowledge, attitude, and practice of COVID-19 precautionary measures and the frequency of infection among medical students at an Egyptian University. PLOS ONE. 2022 Sep 19;17(9):e0274473. doi: 10.1371/journal.pone.0274473 36121862 PMC9484682

[19] Zhang J, Yin Y, Dean J, Zhang X, Zhang Y, Wang J, et al. Knowledge, Attitude, and Practice Survey of COVID-19 Among Healthcare Students During the COVID-19 Outbreak in China: An Online Cross-Sectional Survey. Front Public Health. 2021;9:742314. doi: 10.3389/fpubh.2021.742314 34692628 PMC8528949

[20] Bokemper SE, Cucciniello M, Rotesi T, Pin P, Malik AA, Willebrand K, et al. Experimental evidence that changing beliefs about mask efficacy and social norms increase mask wearing for COVID-19 risk reduction: Results from the United States and Italy. PLOS ONE. 2021 Oct 11;16(10):e0258282. doi: 10.1371/journal.pone.0258282 34634089 PMC8504748

[21] Kohls E, Baldofski S, Moeller R, Klemm SL, Rummel-Kluge C. Mental Health, Social and Emotional Well-Being, and Perceived Burdens of University Students During COVID-19 Pandemic Lockdown in Germany. Front Psychiatry. 2021;12:643957. doi: 10.3389/fpsyt.2021.643957 33889102 PMC8055863

[22] Al-Maskari A, Al-Riyami T, Kunjumuhammed SK. Students academic and social concerns during COVID-19 pandemic. Educ Inf Technol. 2022 Jan 1;27(1):1–21. doi: 10.1007/s10639-021-10592-2 34226817 PMC8243059

