# Supplementary material for: Knowledge, attitudes and practices of French university students towards COVID-19 prevention—are health students better?
Source: PLoS One. 2023 Nov 1;18(11):e0287716. doi: 10.1371/journal.pone.0287716 (PMC10619770; doi:10.1371/journal.pone.0287716)
Supplement: S1 Appendix — (PDF) [file pone.0287716.s001.pdf]

Survey questionnaire

You are presently enrolled at the University of Rouen Normandy. The teaching team at UFR Santé is interested in hearing your thoughts regarding the ongoing Covid-19 outbreak and its impact on public health. To this end, we kindly request that you take 6 minutes to complete an anonymous survey. Your responses will be analysed and used to inform educational initiatives. We assure you that your data will be kept strictly confidential, and we ask that you refrain from providing any personally identifiable information.

We value your individual, independent, and unbiased opinions and appreciate your candid responses. Thank you very much for your participation.

Q1) What is your age group?

☐ Under 20 years ☐ 20-years ☐ 25-30 years ☐ over 30 years

Q2) Are you

☐ a man ☐ a woman

Q2B) Which department are you enrolled in for the current year?

Q2C) Are you a health student doing internships at the hospital?

Q3) In your opinion, what is the positivity rate of Covid-19 PCRs in France at the moment?

A. < 1%
B. 1 to 5%
C. 5 to 10 %
D. > 10 %
E. I don't know

Q4) In your opinion, what is the positivity rate of Covid-19 PCRs in the metropolis of Rouen, currently?

A. < 1%
B. 1 to 5%
C. 5 to 10 %
D. > 10 %
E. I don't know

Q5) In your opinion, what are effective and ineffective actions to address the transmission of COVID-19 infection at the population level (= outside of the hospital setting)?

For each proposition below, choose among: Ineffective / Not very Effective / Moderately Effective / Very Effective / Don't know

SQ001 Regular hand washing with soap and water

SQ002 Regular hydro-alcohol hand friction
SQ003 Do not allow physical contact (don't greet by kissing or shaking hands…)
SQ004 Respect for 1-meter physical distancing
SQ005 Disinfection of any surface before touching it
SQ007 Wearing a cloth mask
SQ008 Wearing a transparent face shield
SQ009 Wearing a surgical mask
SQ0010 Coughing/sneezing into the crook of the elbow
SQ011 Use a single-use handkerchief, discarded after use
SQ012 Isolation of confirmed Covid-19 cases
SQ013 isolation of contacts of confirmed Covid-19 cases
SQ014 Isolation of contacts from persons identified as contacts of confirmed Covid-19 cases (« contacts of contacts »)

Do you think you apply barrier gestures
Q6A) in your personal life?

☐ Perfectly ☐ Pretty good ☐ Pretty Not ☐ Not at all

Q6B) on the premises of the University?

☐ Perfectly ☐ Pretty good ☐ Pretty Not ☐ Not at all

Q6C) in the hospital (if you work there on an internship)?

☐ Perfectly ☐ Pretty good ☐ Pretty Not ☐ Not at all

Q7) Are you aware of a Covid-19 health protocol at the University of Rouen Normandy?

☐ Yes ☐ no

Q7B) If YES,
Do you know its contents?

☐ Yes, absolutely ☐ Yes, Partially ☐ Not at all

Q7C) Are you applying it?

☐ Yes, absolutely ☐ Yes, Partially ☐ Not at all

Q8) At the University, do you wear a mask (surgical mask or cloth mask)?

A. Systematically
B. Regularly
C. Rarely
D. Never

Q9) At the University, do you use hand hygiene (washing and/or rubbing with a hydro-alcoholic solution)?

A. Systematically
B. Regularly

C. Rarely
D. Never

Q10) If you are an internship student at the hospital,
Do you wear a surgical mask

A. Systematically
B. Regularly
C. Rarely
D. Never

Q10B) Do you wear a surgical mask when walking in hospital corridors?

A. Systematically
B. Regularly
C. Rarely
D. Never

Q10C) In the hospital, do you wear a surgical mask while you remain inside an office?

A. Systematically
B. Regularly
C. Rarely
D. Never

Q11. In your situation, what are the possible obstacles to the respect of barrier gestures?

SQ001) I find them too restrictive
SQ002) I think they are useless or not enough useful
SQ004) As my risk of severe Covid-19 is low, I feel little concerned
SQ005) As my surroundings are at low risk of severe Covid-19, I feel little concerned
SQ006) As I don’t have symptom suggestive of Covid-19, I feel little concerned
SQ007) As I have been ill with Covid-19 in the past, I feel little concerned
SQ008) I am aware of the risks, but I make the deliberate choice to expose myself
SQ009) Compliance with these precautionary measures is my personal choice
Other (please specify)

Q12) Since September 1, 2020, have you personally been screened for Covid-19 by PCR?

☐ Yes ☐ No

Q12B) f YES, under what circumstances?
SQ002) following the appearance of evocative symptoms of Covid-19
SQ003) Following a contact with a confirmed Covid-19 case
SQ004) As part of a university screening campaign
SQ005) Personal initiative
Other (please specify)

Q12C) What was the result?

☐ Positive ☐ Negative ☐ I don’t know

Q14) Verbatim
If you wish, you can now make a personal comment about this survey (free comment):

Thank you for your participation!
